# Identification of Photoperiod- and Phytohormone-Responsive DNA-Binding One Zinc Finger (Dof) Transcription Factors in *Akebia trifoliata* via Genome-Wide Expression Analysis

**DOI:** 10.3390/ijms24054973

**Published:** 2023-03-04

**Authors:** Qiuyi Zhang, Shengfu Zhong, Qing Dong, Hao Yang, Huai Yang, Feiquan Tan, Chen Chen, Tianheng Ren, Jinliang Shen, Guoxing Cao, Peigao Luo

**Affiliations:** 1 Key Laboratory of Plant Genetics and Breeding, Sichuan Agricultural University of Sichuan Province, Chengdu 611130, China; 2 College of Forestry, Sichuan Agricultural University, Chengdu 611130, China

**Keywords:** *Akebia trifoliata*, Dof transcription factor, genome-wide duplication, photoperiod, phytohormone responsive, RT-qPCR

## Abstract

As a kind of plant-specific transcription factor (TF), DNA-Binding One Zinc Finger (Dof) is widely involved in the response to environmental change, and as an evolutionarily important perennial plant species, *Akebia trifoliata* is ideal for studying environmental adaptation. In this study, a total of 41 *AktDofs* were identified in the *A. trifoliata* genome. First, the characteristics, including the length, exon number, and chromosomal distribution of the *AktDofs* and the isoelectric point (PI), amino acid number, molecular weight (MW), and conserved motifs of their putative proteins, were reported. Second, we found that all *AktDofs* evolutionarily underwent strong purifying selection, and many (33, 80.5%) of them were generated by whole-genome duplication (WGD). Third, we outlined their expression profiles by the use of available transcriptomic data and RT-qPCR analysis. Finally, we identified four candidate genes (*AktDof21*, *AktDof20*, *AktDof36*, and *AktDof17*) and three other candidate genes (*AktDof26*, *AktDof16*, and *AktDof12*) that respond to long day (LD) and darkness, respectively, and that are closely associated with phytohormone-regulating pathways. Overall, this research is the first to identify and characterize the *AktDofs* family and is very helpful for further research on *A. trifoliata* adaptation to environmental factors, especially photoperiod changes.

## 1. Introduction

Plants have conspicuous organism responses to environmental factors, and they have even evolved sophisticated specific response mechanisms to several abiotic factors, such as light [1,2]. This indicates that there are some plant-specific genes and regulatory elements and that interactions occur between them [3].

As important regulatory elements, transcription factors (TFs) widely exist in all organisms and regulate various biological processes and metabolic activity [4]. However, numerous studies have confirmed that some TFs, such as DNA-Binding One Zinc Finger (Dof) [5], are plant specific, the concept of which has attracted the attention of many scientists [6]. Dof TFs belong to the zinc finger protein superfamily, and structurally they are usually composed of 200–400 amino acids (AA) and contain two main domains: a highly conserved DNA-binding domain and transcriptional regulatory domains at the N-terminus and the C-terminus, respectively. They are named after the C_2_-C_2_ single-zinc finger structure at the N-terminus, which is composed of 52 AA, so the structure is also called the Dof domain [7]. The covalent binding between four highly conserved cysteine (Cys) residues and Zn^2+^ is the hallmark of Dof TFs. In contrast, the amino acid sequences of the transcriptional regulatory domains at the C-terminus largely vary, which could be responsible for different response mechanisms in plants to various abiotic factors [8].

Since the first *ZmDof1* was identified in maize in 1993 [9], *Dof* genes have been identified and cloned in algae and almost all plant types, including unicellular algae such as *Chlamydomonas reinhardtii* [10], bryophytes such as *Gonium pectorale* [11], ferns such as *Selaginella moellendorffii* [12], and higher plants such as *Amborella trichopoda* [13], which suggests that *Dof* genes are also widespread among plants. Several further studies on both the number and the components of *Dof* genes in various species showed that the *Dof* genes experienced several expansion events throughout plant evolution [13].

In terms of their function, *Dof* genes participate in various plant biological processes, such as responses to both biotic and abiotic stresses. For example, *Dof* genes are involved in the resistance against viruses in tobacco [14] and pepper [15] and fungi in cucumber [16]. At the same time, *Dof* genes are also involved in the response to various abiotic factors, such as photoperiod and temperature, and to different developmental events, such as flowering [17], seed development [18], and leaf senescence [15]. The response processes are usually associated with the regulation of phytohormones, which further suggests that the *Dof* genes could also be involved in regulation by phytohormones [19]. Various studies have shown that plant morphogenesis, photosynthesis, and developmental changes are largely influenced by light (daylength) as well as light quality [20]. In addition, plant perception of changes in daylength is closely associated with the phytohormone response.

*Akebia trifoliata* (*A. trifoliata*) is a woody perennial climbing vine and a member of the Lardizabalaceae family [21]. There is a long history of *A. trifoliata* being widely used as a traditional medicinal plant in East Asia, such as in China [21], Japan [22], and Korea [23]. In the past 30 years, people in many regions have also begun to grow this species as a new fruit crop, and this progress is accelerating due to newly screened cultivars by molecular marker-assisted selection [24]. In fact, *A. trifoliata* is also prized for theoretical studies in addition to commercial exploitation because of its small genome size [25], enough available seeds produced from a single cross, plethora of discernable interesting traits, and short juvenile phase compared with those of other woody tree species [26]. For example, *A. trifoliata* has been widely used in the study of flower development [27], early eudicot evolution [25], and secondary metabolism [28]. Therefore, identifying TFs and further assigning them to their corresponding biological processes are very helpful for promoting the use of *A. trifoliata* for widespread biological studies.

To date, only MADS-box [27] and WRKY TFs [29] have been systemically reported at the genome-wide level, while information about the *Dof* gene family is still elusive. The objectives of this study were to provide an overview of *A. trifoliata Dof* gene family characteristics, including gene structures, chromosomal positions, cis-acting elements, conserved protein motifs, and phylogenetic relationships, by a genome-wide analysis to outline the expression profiles of various fruit tissues at different stages through analysis of the available transcriptomic data and to identify *Dof* genes involved in the response to photoperiod and hormone treatment by the use of RT-qPCR. This study provides basic reference information about *AktDofs* and some useful *AktDofs’* sources for further research on *A. trifoliata* adaptation to changes in environmental conditions, such as daylength.

## 2. Results

### 2.1. Systemic Characterization of the Dof Gene Family in A. trifoliata

We identified a total of 41 *Dof* genes in the *A. trifoliata* genome through a hidden Markov model (HMM) analysis. They were sequentially named *AktDof1*–*41* (Table 1) according to their positions on the chromosome, and their average length was 2048 bp, which varied from 488 bp to 12257 bp. Structurally, *AktDofs* typically contained low numbers of exons, which varied from only one to three; 18 (43.9%) and 19 (46.3%) members had one and two exon(s), respectively. The average PI, amino acid number, and molecular weight (MW) of putative proteins were 8.08, 300 AA, and 33.22 kDa, the ranges of which were 4.73 to 9.48, 150 to 522 AA, and 16.85 to 57.1 kDa, respectively. At the same time, all proteins putatively encoded by the *AktDofs* were spatially located in the nucleus. In terms of evolution, intraspecies collinearity analysis revealed that *AktDofs* were possibly produced by tandem, dispersed and whole (segmental)-genome duplications, but the majority (33, 80.5%) were derived by whole-genome duplication (WGD) events. Physically, most (39, 95.1%) of these genes were unevenly distributed across nearly all 16 *A. trifoliata* chromosomes except chromosome 16, while only *AktDof40* and *AktDof41* were assigned to Contig00776 and Contig01043 of the published *A. trifoliata* reference genome, respectively. Further sliding window analysis with a 250 kb size revealed that 35 out of 41 *AktDofs* were singletons, and only 6 were in 3 clusters (*AktDof4* and *AktDof5* on chromosome 2, *AktDof12* and *AktDof13* on chromosome 4, and *AktDof33* and *AktDof34* on chromosome 13) (Figure S1 (Supplementary Materials)).

### 2.2. Classification of the Dof TFs in A. trifoliata

**Figure 1:**
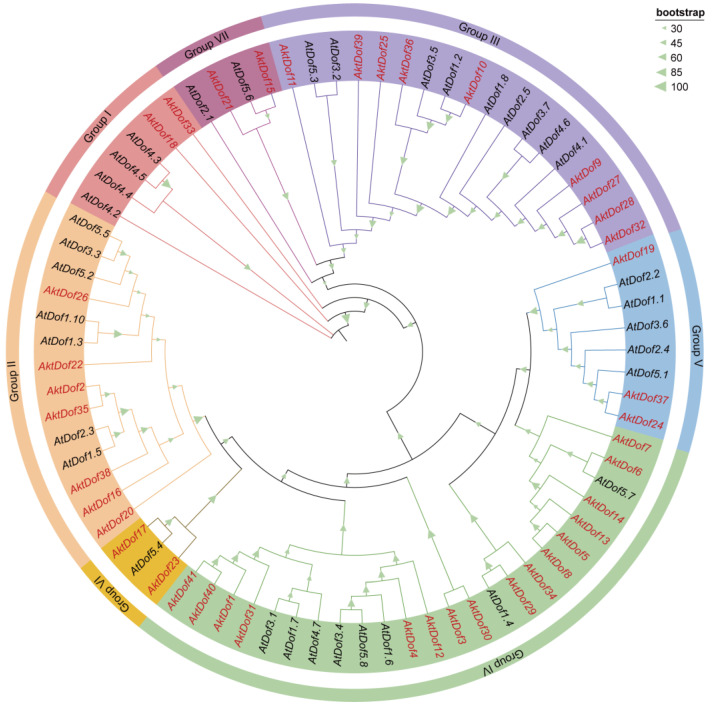
Phylogenetic tree of Dof families in *A. trifoliata* and *Arabidopsis*. Phylogenetic trees were constructed from 77 Dof proteins from *A. trifoliata* (41) and *Arabidopsis* (36) using the ML method and 1000 bootstrap replicate. Different branch colors and background colors represent seven groups. AktDofs is indicated in red, AtDofs in black, the bootstrap program is displayed in the green triangle on the branch.

To classify the *AktDof* gene family, 41 AktDofs proteins and 36 reference *Arabidopsis* Dof proteins (AtDofs) together were used to construct a phylogenetic tree (Figure 1). The results showed that the 77 *Dof* proteins could be divided into 7 groups, namely, group I (2 AktDofs and 4 AtDofs), group II (7 AktDofs and 7 AtDofs), group III (9 AktDofs and 9 AtDofs), group IV (16 AktDofs and 8 AtDofs), group V (3 AktDofs and 5 AtDofs), group VI (2 AktDofs and 1 AtDofs), and group VII (2 AktDofs and 2 AtDofs). Although, the AktDofs could be broadly divided into all these groups and even all the subgroups, the homology within the AktDofs was higher than that between the AktDofs and AtDofs.

### 2.3. Gene Structure, Conserved Motifs and Phylogenetic Tree of AktDofs

**Figure 2:**
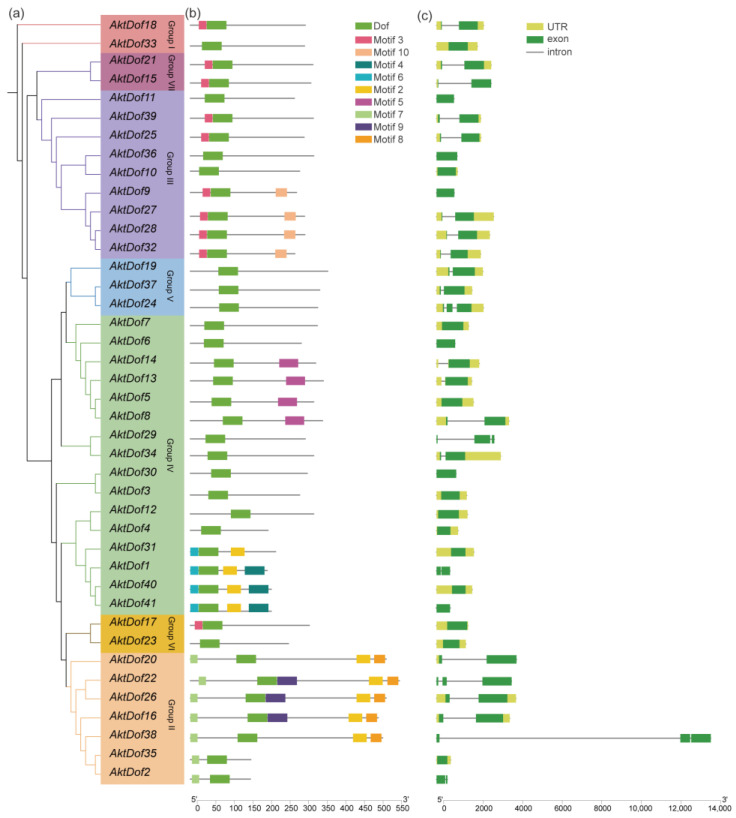
Phylogenetic relationship, conserved motif analysis, and gene structure of *AktDofs*. (**a**) Phylogenetic tree of 41 AktDof proteins. (**b**) Conserved motifs of the AktDofs protein. (**c**) Exon-intron structure of the *AktDofs*. The 10 top conserved motifs are shown in different colored boxes. The green boxes represent exons, the black lines represent introns, and the upstream/downstream regions of the *AktDofs* are shown in yellow.

According to the distribution and evolutionary relationship of the phylogenetic tree of both the *A. trifoliata* and *Arabidopsis* Dof protein (Figure 1), we divided 41 AktDofs into 7 groups: group I (2 AktDofs), group II (7 AktDofs), group III (9 AktDofs), group IV (16 AktDofs), group V (3 AktDofs), group VI (2 AktDofs), and group VII (2 AktDofs) (Figure 2). The Dof motif widely exists in all the 41 AktDofs, whereas several other motifs appear only in one group. For example, motifs 4, 5, and 6 exist only in group IV; motifs 8 and 9 exist only in group II; and motif 10 exists only in group III. The number of genes containing the remaining 3 motifs vary from 7 to 10. The number of motifs in the same group ranged from 1 to 5, and the number of motifs is the largest in group II. These findings suggested that the genes in group II may have undergone a longer evolutionary history. The details of all motifs are shown in Table S1.

The exon–intron structure was relatively similar in each group, but there was no obvious correlation between exon number and gene length. *AktDofs* usually contain a relatively long exon region, which may be the main region that performs gene functions, and the rest of the exons are shorter in length. Noticeably, *AktDof38* has the longest intron with 10817 bp. Six *AktDofs* (*AktDof11*, *AktDof41*, *AktDof9*, *AktDof36*, *AktDof30*, and *AktDof6*) contained only exons and lacked both introns and untranslated regions (UTRs).

### 2.4. Ka/Ks Value of Homologous AktDof Pairs

The Ka/Ks values of all 439 homologous *AktDof* pairs were much lower than 1 and varied from 0 (*AktDof41* and *AktDof1*, *AktDof1* and *AktDof40*) to 0.67 (*AktDof1* and *AktDof38*) (Table S2), which indicated that *AktDof1* could be an ancestral gene of the *AktDof* family. Moreover, there were only five homologous *AktDof* gene pairs with Ka/Ks values larger than 0.5, indicating that the *AktDofs* could have experienced a strong purifying selection during their evolutionary history.

### 2.5. Interspecies Collinearity of AktDofs

To determine the collinearity of *AktDofs* among several main evolutionary clades of angiosperms, the reference genomes of two basal eudicots (*A. trifoliata* and *Papaver somniferum*), three core eudicots (*Arabidopsis thaliana*, *Coffea arabica*, *Malus domestica*) and three monocots (*Brachypodium distachyon*, *Setaria viridis*, *Zea mays*) were used to search for *Dof* genes, and only *Dof* genes mapped on assembled chromosomes were further used for interspecies collinearity analysis. We found 39, 36, 32, 50, 28, 36, and 46 *Dof* genes in the *P. somniferum*, *A. thaliana*, *C. arabica*, *M. domestica*, *B. distachyon*, *S. viridis*, and *Z. mays* genomes, respectively. Then, we detected 86, 54, 78, 98, 21, 27, and 30 homogeneous gene pairs between *AktDofs* and 36 *PsDofs*, 26 *AtDofs*, 31 *CaDofs*, 41 *MdDofs*, 12 *BdDofs*, 16 *SvDofs*, and 18 *ZmDofs*, respectively (Figure S2, Table S3). In addition, *AktDof14* had corresponding homogenous genes only in the basal eudicots, and both *AktDof20* and *AktDof38* had corresponding homogenous genes in the basal eudicot *P. somniferum* and all three monocots but not in any of the three core eudicots. Likewise, 27 out of 39 *AktDofs* had corresponding homogenous genes in the basal eudicot *P. somniferum* and all three core eudicots but did not have them in any of the three monocots.

### 2.6. Identification of Cis-Acting Elements of the AktDof Gene Family

**Figure 3:**
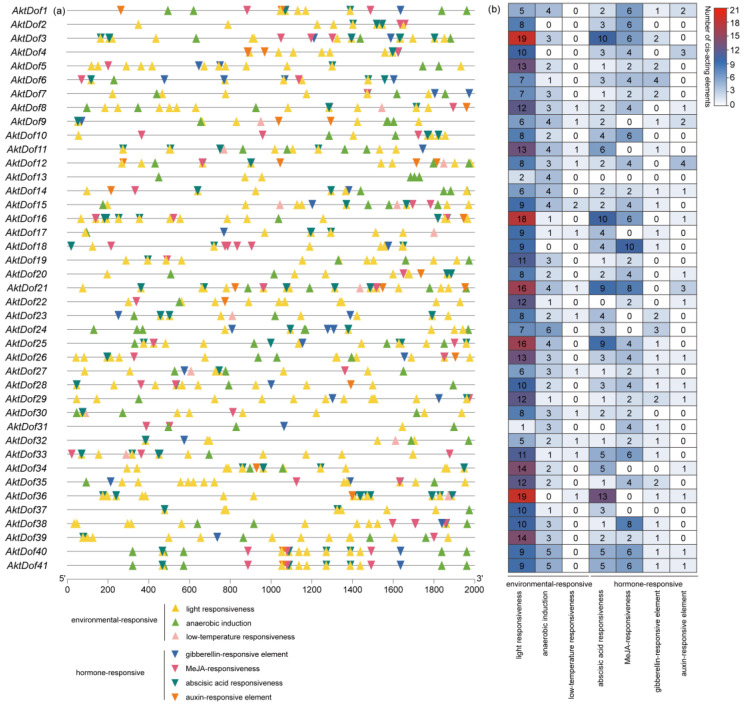
Visualization of transcription factor-binding sites (TFBSs) within the promoter region of *AktDofs*. (**a**) Two kilobase region upstream of the transcription start site of the *AktDofs*. (**b**) The number of cis-acting elements of the two functional categories in the *AktDofs* is represented by different colors and numbers.

Both the type and the number of cis-acting elements within the 2000 bp upstream regions of the start codon of the *AktDofs* are listed in Figure 3, which shows that the types of cis-acting elements included hormone-responsive and environment-responsive elements, each with four and three subtypes, respectively. The hormone-responsive types included methyl jasmonate (MeJA)-responsive (CGGTA motif-containing, TGACG motif-containing), abscisic acid (ABA)-responsive (ABRE), gibberellin (GA)-responsive (TATC-boxes, P-boxes, GARE motif-containing), and auxin-responsive (AuxRR-core, TGA) elements, while the environment-responsive types included anaerobic induction (ARE)-responsive, light-responsive (G-box), and low-temperature-responsive (LTR) elements. In addition, a total of 870 cis-acting elements of the *AktDofs* were identified, and there were 342 and 528 hormone-responsive and environment-responsive elements, respectively. Further comparison showed that the numbers of light-responsive and low-temperature-responsive elements were the highest (410) and the lowest (14), respectively.

Both the type and the number of cis-acting elements also widely varied among members of the *AktDof* genes (Table S4). We found that every *AktDof* gene had a light-responsive element with numbers ranging from 1 to 19, while no *AktDof* included any of the 7 subtypes; *AktDof21* and *AktDof13* had the most (41) and least (6) cis-acting elements, respectively; the number of cis-acting element subtypes varied from 2 (only in *AktDof13*) to 6; and 2, 9, 13, and 16 genes contained three, four, five and six cis-acting element subtypes, respectively.

### 2.7. Expression Profiles of AktDofs in A. trifoliata Tissues at Various Stages

Among the 41 *AktDofs*, 40 exhibited detectable expression and only the expression of *AktDof1* could not be detectable in any samples, although most of them had low expression in three different fruit tissues (peel, flesh, and seed tissues) at four periods (young, enlargement, coloring, and mature stages) as determined by the analysis of the available transcriptomic data (Figure S3). The highest expression level occurred for *AktDof12* in the first stage of the seeds (fragments per kilobase of transcript per million mapped reads (FPKM = 42.69), and the lowest expression was detected for *AktDof16* (FPKM = 0.38 × 10^−2^) in the fruit flesh at the fourth stage. Only *AktDof28* in the fruit flesh at the fourth stage, *AktDof17* in the peel at the first stage, and *AktDof12* in the seed at the first stage were identified as having medium-high expression (FPKM > 30). *AktDof12* and *AktDof7* were almost exclusively expressed in the seeds, but the expression of *AktDof12* gradually decreased as the fruit matured, while the expression of *AktDof7* gradually increased.

**Figure 4:**
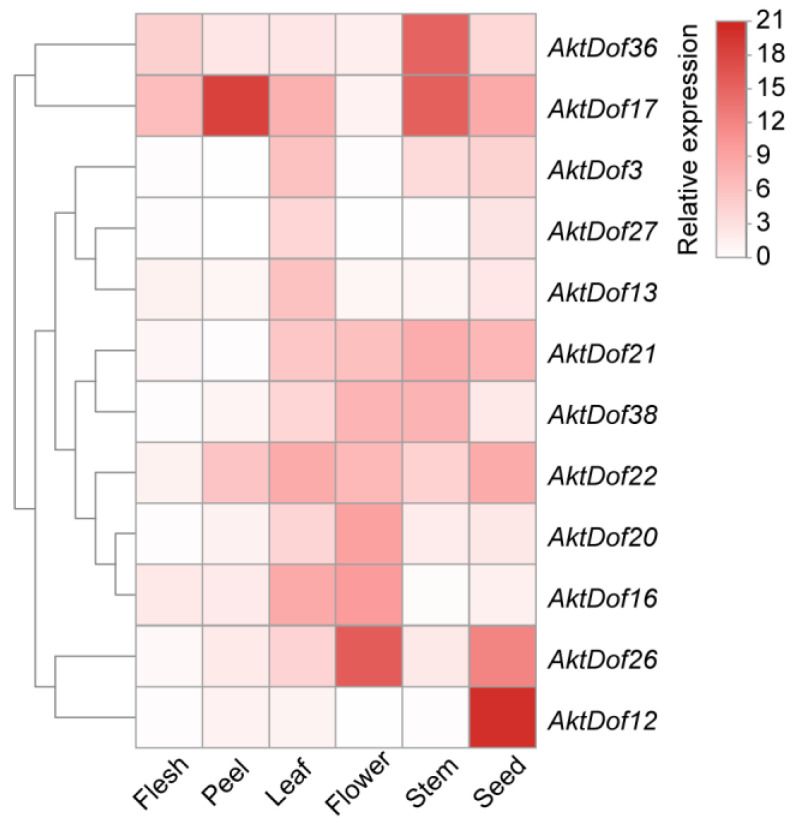
Expression profiles of 12 *AktDofs* in different tissues. The variation in the degree of white to red indicates the intensity of the expression level. The calculation of the relative expression level was based on that of the *GAPDH* gene as the internal reference, and the expression level of the gene was calculated by the 2^−ΔΔCt^ method.

In addition, we screened 12 *AktDofs* of which 5, 2, 3, 1, and 1 were members of groups II, III, IV, VI, and VII, respectively, according to the number and putative responsive type of their cis-acting elements (Figure 3). Then, we measured the expression levels of these 12 *AktDofs* in the leaves, flowers, stems, fruit peels, fruit flesh, and seeds by RT-qPCR. The results showed that *AktDof36* in the stems, *AktDof17* in the fruit peels and stems, *AktDof26* in the flowers and seeds, and *AktDof12* in the seeds exhibited high expression. *AktDof12* exhibited the highest expression in the seeds and the lowest expression in other parts, which is consistent with the transcriptomic data. All 12 genes were expressed to a lesser degree in the peel and to a great degree in the leaves and seeds (Figure 4).

### 2.8. Effects of Exogenous Hormones on the Expression of AktDofs

**Figure 5:**
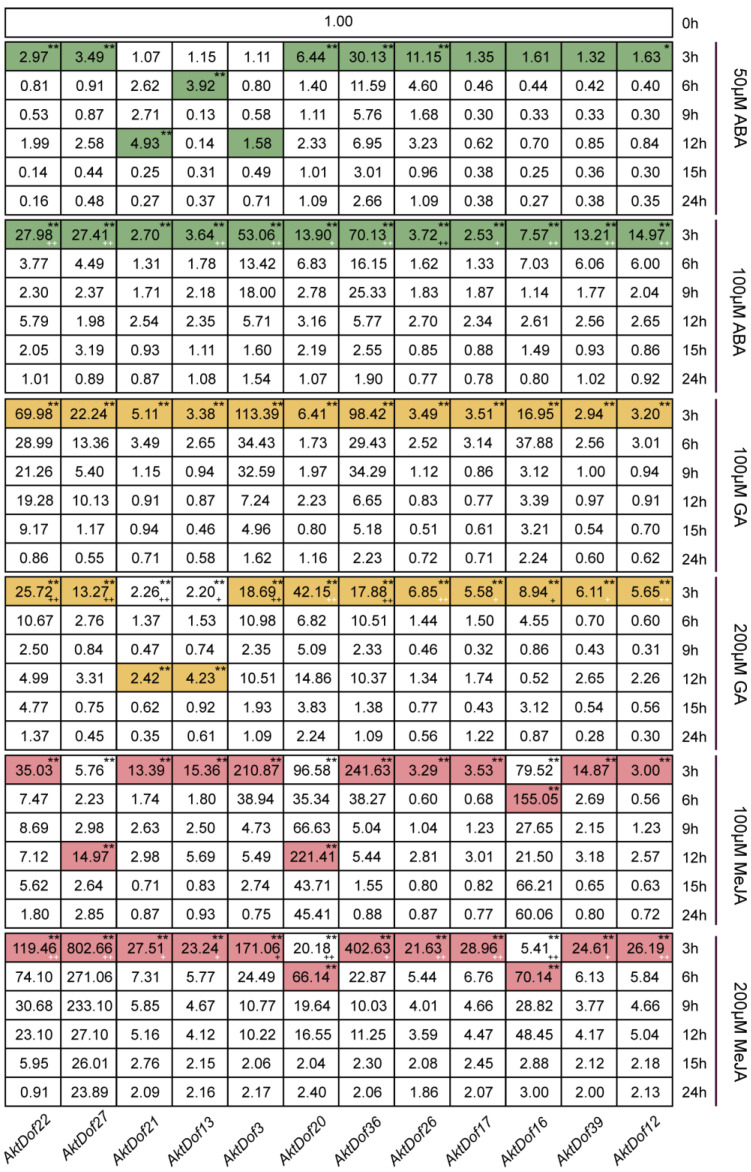
Expression patterns of 12 *AktDofs* in leaves in response to three hormone treatments. Colored undertones represent the expression peaks, and the different colors represent the different hormones. * represents the significance between both peak expression and the expression at 3 h and 0 h (*, *p* < 0.05; **, *p* < 0.01), + represents the significance between two concentrations of each hormone, + (white) indicated high-concentration hormone expression is significantly higher than low-concentration hormone expression while + (black) is the opposite, (+, *p* < 0.05; ++, *p* < 0.01).

The RT-qPCR results showed that almost all 12 genes significantly responded to six treatments, namely, ABA (50 μM and 100 μM), GA (100 μM and 200 μM), and MeJA (100 μM and 200 μM) (Figure 5), and the responses of only *AktDof3*, *AktDof17*, *AktDof16*, and *AktDof38* to 50 μM ABA were not significant. In addition, many genes showed a relatively strong response to the high levels of ABA (100 μM) and MeJA (200 μM) and a stronger response to the low concentration of GA (100 μM). Finally, the response was very fast, and almost all peak expression values occurred after 3 h of treatment with the exogenous hormones; the exceptions were the peak expression values for *AktDof21* (at 12 h) in response to 50 μM ABA, *AktDof21* and *AktDof13* (at 12 h) in response to 200 μM GA, and *AktDof27* and *AktDof20* (at 12 h) as well as *AktDof16* and *AktDof20* (at 6 h) in response to 100 μM MeJA. Almost all gene expression levels gradually decreased and ultimately returned to normal levels after 24 h.

### 2.9. Differential Expression of AktDofs in Response to Photoperiod Treatments

**Figure 6:**
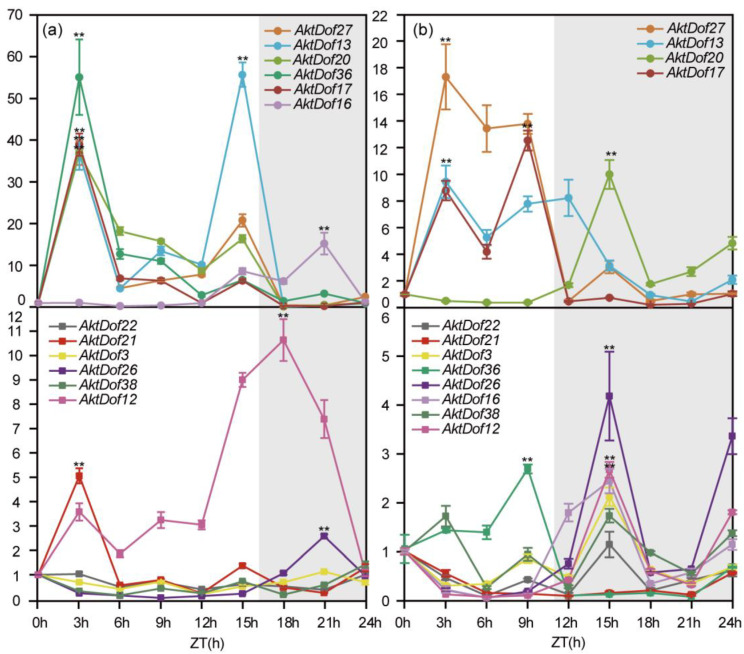
Expression patterns of 12 *AktDofs* in leaves in response to different daylengths. (**a**) The LD treatment. (**b**) The SD treatment. Time (h) is expressed as hours from dawn (ZT, zeitgeber), and the light gray represents night. The error bars represent the standard deviations of three replicates. * represents the significance between peak expression and the expression at 0 h (**, *p* < 0.01).

The results showed that the 12 *AktDofs* could be divided into two types (those expressed at a high level and those expressed at a low level) according to their highest expression under both long day (LD) and short day (SD) conditions, and for clarity, we artificially set the thresholds to 16 h and 11 h for LD and SD conditions, respectively (Figure 6). All the data and results of the statistical analysis are shown in Figure S4. Under LD conditions, the highly expressed genes included *AktDof27*, *AktDof13*, *AktDof20*, *AktDof36*, *AktDof17*, and *AktDof16*, and all six of these genes exhibited a significant difference at the *p* = 0.05 level between the LD and SD conditions. There was one gene (*AktDof16*) whose expression peaked at 21 h (dark), and the expression of the remaining five genes was highest at 3 h (light) (*AktDof27*, *AktDof20*, *AktDof36*, *AktDof17*) or at 15 h (light) (*AktDof13*). Likewise, the genes that were expressed at a low level included *AktDof22*, *AktDof21*, *AktDof3*, *AktDof26*, *AktDof38*, and *AktDof12*, of which the expression of *AktDof22*, *AktDof3*, and *AktDof38* was very low and of which the expression difference between the LD and SD conditions was very small and not significant at the *p* = 0.05 level. The peak gene expression of *AktDof21* and *AktDof12* occurred at 3 h (light), while that of *AktDof26* occurred at 18 h (dark).

Under SDs, the highly expressed genes included *AktDof27*, *AktDof13*, *AktDof20*, and *AktDof17*, and all four genes exhibited a significant difference between LD and SD conditions at the *p* = 0.05 level. *AktDof20* and *AktDof17* were expressed the most at 21 h (dark) and 9 h (light), respectively. The expression peaks of both *AktDof27* and *AktDof13* occurred at 3 h (light). Additionally, the genes expressed at a low level included *AktDof22*, *AktDof21*, *AktDof3*, *AktDof36*, *AktDof26*, *AktDof16*, *AktDof38*, and *AktDof12*, among which the expression of *AktDof22*, *AktDof21*, *AktDof3*, and *AktDof38* was very low, and the expression difference between the LD and SD conditions was not significant at the *p* = 0.05 level. There was one gene (*AktDof36*) whose expression peaked at 9 h (light), and *AktDof26*, *AktDof16*, and *AktDof12* were expressed highest at 15 h (dark).

In total, we found that *AktDof22*, *AktDof3*, and *AktDof38* expression did not change significantly under different daylengths; *AktDof27*, *AktDof13*, *AktDof36*, and *AktDof17* expression was induced by light under both LD and SD conditions; and *AktDof26*, *AktDof16*, and *AktDof12* expression was higher in the dark. *AktDof20* exhibited an opposite circadian expression pattern under the different daylength. *AktDof21* was not expressed under SDs, but its expression was found to be induced by light under LD.

## 3. Discussion

As plant-specific TFs, *Dof* genes widely participate in plant responses to environmental changes and are also closely associated with pathways regulated by hormones. *A. trifoliata* is a typical woody perennial species that has evolved a sophisticated strategy to adapt well to environmental variation. In addition, the available reference genome and transcriptomic data afford a chance to identify and screen *Dof* genes involved in the response of *A. trifoliata* to photoperiods and exogenous hormones.

### 3.1. AktDof Gene Family Members May Have Evolutionarily Diverged

Similarity searches of all predicted proteins showed that 65–85% of all *Arabidopsis* genes are homologous to at least one other gene in the genome [30], which suggested that gene families are ubiquitous in the plant genome. Genes duplicated by different mechanisms such as WGD, tandem, and dispersed duplications, are primary raw materials for new gene origins and evolution and ultimately result in functional novelty and specialization [31]. Some studies have shown that following WGD, genes encoding TFs are preferentially retained [32].

In this study, 33 (80.5%) of 41 identified *AktDofs* were found to be derived from WGD events (Table 1), which suggested that WGD could be the major force of *AktDof* origin. In addition, the fact that all Ka/Ks values of the homologous *AktDof* pairs were much lower than 1 (Table S2) further suggested that all *AktDofs* experienced a strong purifying selection during their evolutionary history. In addition, the Ka/Ks value of two combinations between *AktDof1* and both *AktDof41* and *AktDof40* was very close to zero, while the combination (*AktDof1* and *AktDof38*) with the largest Ka/Ks value was also related to *AktDof1* (Table S2), which indicated that *AktDof1* could be an ancestral gene of the *AktDof* family.

### 3.2. The functions of AktDofs Could Largely Differ

As we know, gene characteristics such as structure [33], expression profiles [17], and evolutionary events experienced [34] could be highly associated with gene functions, which indicated that gene function diversity could be weighed by the difference in their characters. In terms of their structure, the 41 identified *AktDofs* first largely differed in their length, putative protein PI, amino acid number, and MW (Table 1). Although the exon number varied little, the exon length also largely differed. Second, both the number and the components of the conserved motifs were obviously different (Figure 2, Table S1), and the results of the phylogenetic analysis revealed that the members of the *AktDof* gene family were present in all seven of the resulting groups (Figure 2). Third, the number, type, and components of cis-acting elements of the 41 *AktDofs* also largely varied (Figure 3). Obviously, large structural differences would potentially lead to their functional differences.

With respect to the expression profiles, our transcriptomic data analysis revealed that some *AktDofs*, such as *AktDof11*, *AktDof15*, *AktDof2*, *AktDof1* and *AktDof6*, exhibited very low expression, while some *AktDofs*, such as *AktDof27*, *AktDof20*, *AktDof26*, *AktDof28*, and *AktDof17*, exhibited high expression. In addition, *AktDof33*, *AktDof7*, and *AktDof12* had seed-specific expression patterns, while *AktDof34*, *AktDof5*, and *AktDof32* had developmental stage-specific patterns. The RT-qPCR results also confirmed that tissue-specific expression largely occurred for the 12 selected *AktDofs*. The *AktDof* expression profiles and patterns indicated that different *AktDofs* had different biological functions.

In terms of evolution, different duplication types provide the possibility for the same members of a gene family to have different structures and functions. In the present study, intraspecies collinearity analysis revealed that *AktDofs* experienced multiple duplication types, including tandem, dispersed, and WGD types, although the majority (33, 80.5%) were derived from WGD events. In addition, all *AktDofs* underwent purifying selection, but the Ka/Ks values still exhibited large variations 0 to 0.67 (Table S2). Obviously, both the duplication type and the selection strength of *AktDofs* indicated that they perform different functions. Overall, the large difference of *AktDofs* in structure, expression pattern, and evolutionary history suggested that they would also have had a different function.

### 3.3. Some Putative Candidate Genes Are Involved in the Response to Photoperiod

In plants, the response to changes in the photoperiod is a key factor regulating the transition from vegetative to reproductive development. Various studies have shown that *Dof* genes are widely involved in the light response in *Arabidopsis* [35], pea [36], rice [37] and tomato [38]. In recent years, most of the functional studies on *Dof* genes have been carried out in *Arabidopsis*, and most of the homologous genes have similar functions.

In this study, nearly all the 12 studied genes significantly responded to three exogenous hormones (Figure 5), which indicated that they could have versatile functions in pathways associated with phytohormones. By performing an RT-qPCR analysis, we found that *AktDof22*, *AktDof3*, and *AktDof38* exhibited very low expression under both LD and SD conditions and that there was no significant difference between LD and SD conditions. Hence, these three genes could not be involved in the response process to the photoperiod. Four genes, namely, *AktDof21*, *AktDof20*, *AktDof36*, and *AktDof17*, exhibited highly significantly increased expression levels at 3 h under LD conditions, which indicated that they could be closely associated with changes in the photoperiod and induced by light because their expression under LD conditions was obviously different from that under SD conditions. Likewise, *AktDof26*, *AktDof16*, and *AktDof12* exhibited obviously increased expression levels in the dark compared with the light under both LD and SD conditions, which suggested that they could be induced by darkness. The primary results afforded useful clues to further study the function of *AktDofs* by other molecular methods such as complementary analysis in the future though they could not provide directly solid evidence for the function. 

## 4. Materials and Methods

### 4.1. Identification of Dof TFs in A. trifoliata

Genome sequence annotation files (accession IDs: SAMN16551931-33) were downloaded from the National Center for Biotechnology Information database (NCBI) (https://www.ncbi.nlm.nih.gov/bioproject/PRJNA671772; accessed on 20 April 2022) under BioProject ID PRJNA671772. The Dof domain zinc finger (PF02701) sequence from the Pfam database was downloaded to identify Dof TFs, and then the sequences of all the proteins of *A. trifoliata* were scanned by HMMER 3.0 using the HMM with an E value of 1 × 10^−5^. We subjected the gene sequences obtained in the above steps to SMART (http://smart.embl.de/smart/batch.pl, accessed on 2 May 2022) and the NCBI Conserved Domain Database (CDD) (https://www.ncbi.nlm.nih.gov/cdd/, accessed on 2 May 2022) to remove redundant and non-conserved genes. Then, the resulting 41 *Dof* candidate genes that were screened were named *AktDof1–41*. The AktDof protein sequences are listed in Table S5. The corresponding general feature format (GFF3) file was used to anchor the chromosome locations of the *AktDofs* and to map their physical locations on the chromosomes. To determine the *AktDof* clusters on the chromosomes, we used a sliding window size of 250 kb. MCScanX and TBtools [39] software were used for intraspecies collinearity analysis and gene duplication event analysis, respectively. The protein MW and PI were computed via ExPASy (https://web.expasy.org/compute_pi/, accessed on 16 May 2022), and the protein subcellular localization was predicted by Cell-Ploc (http://www.csbio.sjtu.edu.cn/bioinf/plant-multi/, accessed on 16 May 2022).

### 4.2. Sequence Characteristic Analysis, Phylogenetic Analyses, and Collinearity of AktDofs

Multiple alignments of the full-length protein sequences were executed by using ClustalW (https://www.genome.jp/tools-bin/clustalw, accessed on 20 May 2022). A phylogenetic tree was constructed using MEGA 11 software (version 11.0.10) via the maximum likelihood (ML) method with 1000 bootstrap replicates. The GFF3 file of the *A. trifoliata* genomic annotation was used to analyze the gene sequence characteristics. Gene Structure Display Server (http://gsds.gao-lab.org/, accessed on 20 May 2022) was used to count the number and location of exons/introns of the *AktDofs*. The conserved motifs of the *A. trifoliata* proteins were analyzed by MEME Suite (https://meme-suite.org/meme/tools/meme, accessed on 20 May 2022), where the maximum motif number was set to 10 and the other settings were set to their default values. The above results were subsequently visualized by TBtools [39] software (version 1.0876). To display the evolutionary selection pressure between collinear gene pairs, the Ka/Ks ratio was calculated by the TBtools [39] software (version 1.0876). The reference genome sequences of *P. somniferum* (accession ID: PRJNA435796), *A. thaliana* (accession ID: PRJDB14952), *C. arabica* (accession ID: PRJNA497895), *M. domestica* (accession ID: PRJNA339703), *B. distachyon* (accession ID: PRJNA32607), *S. viridis* (accession ID: PRJNA265547), and *Z. mays* (accession ID: PRJEB32225) were downloaded from the NCBI database and were used to perform a collinearity analysis with the sequence of *A. trifoliata*. A phylogenetic tree comprising 36 *AtDofs* and 41 *AktDofs* was constructed using MEGA 11 software (version 11.0.10) by the ML method with 1000 bootstrap replicates. The PlantCARE online website (https://bioinformatics.psb.ugent.be/webtools/plantcare/html/, accessed on 26 May 2022) was then used to analyze the cis-acting elements in the 2000 bp promoter region upstream of *A. trifoliata* [40].

### 4.3. Expression Analysis of AktDofs in Different Fruit Tissues and at Different Developmental Stages of A. trifoliata

The transcriptomic data of *A. trifoliata* were downloaded from the NCBI database under BioProject ID PRJNA671772 (https://www.ncbi.nlm.nih.gov/bioproject/PRJNA671772; accessed on 25 April 2022). The *A. trifoliata* transcriptomic data contained data on three tissue types (fruit flesh, seeds, fruit peels) at four different stages (young, enlargement, coloring, and mature stages), and there were data for three biological replicates (young stage, SAMN16551934-36, enlargement stage; SAMN16551937-39, coloring stage; SAMN16551940-42, mature stage). FPKM values calculated by HISAT2 and DESeq2 were used to estimate gene expression levels.

Tissues from sequenced ‘Shusen 1′ plants were randomly selected to measure gene expression in different organs via the quantitative RT-PCR (RT-qPCR). Leaves, stems, and mixed flower samples (which included male and female flower buds) were collected at the rapid-growth stage (approximately 10 days before flowering), after which the flesh, peels, and seeds were harvested at the mature stage and then stored at −80 °C until RNA isolation. Three biological replicates were collected. Finally, TBtools (version 1.0876) [39] software was used to construct a heatmap of *AktDofs*’ expression.

### 4.4. Expression Patterns of AktDofs in Response to Different Hormone Treatments and Different Photoperiods

To investigate the *AktDofs*’ response to hormones, different exogenous hormone treatments were applied. The cuttings used for the experimental treatment were obtained from the same tree cuttings and were exposed to the same cultivation conditions. The cuttings were transplanted in the germplasm nursery of the Sichuan Agricultural University Chongzhou Research Station (30°43′ N, 103°65′ E); each cutting had 10 or more new leaves, at which point they were considered suitable for use as experimental materials. For the exogenous hormone-treated group, the roots were exposed to gibberellin (GA) (200 μM and 100 μM), abscisic acid (ABA) (50 μM and 100 μM), and methyl jasmonate (MeJA) (100 μM and 200 μM) for 3, 6, 9, 12, 15, and 24 h. For the photoperiod treatment group, the cuttings were subjected to short day (SD) and long day (LD) conditions for 5 days, and the young leaves were taken every 3 h from 7:00 am on the sixth day to 7:00 am on the seventh day. Replicates of three different plants were harvested for all treatments, quickly frozen in liquid nitrogen and stored at −80 °C until RNA isolation. The expression of the 12 screened *AktDofs* in the young leaves subjected to LD and SD conditions was measured by RT-qPCR. The relative expression of the genes was calculated using the *GAPDH* gene as an internal reference and the expression of a sample at 0 h as a calibration sample; the 2^−ΔΔCt^ method was used to calculate the expression level of genes. Statistical analysis was performed with SPSS (version 20.0.0) and Origin 2018 software (version 9.5.1).

### 4.5. RNA Isolation and RT-qPCR

Total RNA was extracted with an RNAprep Pure Plant Plus Kit (Polysaccharides and Polyphenolics-rich) (TIANGEN, Beijing, China). The integrity and purity of the RNA were checked via an Agilent 2100 Bioanalyzer (Agilent Technologies, Santa Clara, CA, USA) and a NanoDrop ND-1000 spectrophotometer (Thermo Scientific, Austin, TX, USA), respectively. Then, the RNA of the samples was reverse transcribed into cDNA using an EasyScript One-Step gDNA Removal and cDNA Synthesis Supermix Kit (TransGen Biotech, Beijing, China). The primer pairs for the *AktDofs* and *GAPDH* gene were designed using Primer 3.0, and the primer sequences and related details are listed in Table S6. The amount of cDNA was 1μmol as the amplification substrate, and the reaction was carried out as follows: 92 °C for 30 s, followed by 45 cycles of 5s at 92 °C, and 30 s at 60 °C. To determine the expression patterns of the *AktDofs*, RT-qPCR was conducted on a Thermal Cycler CFX96 Real-Time System (Bio-Rad Laboratories, Hercules, CA, USA) together with PerfectStart Green qPCR SuperMix (TransGen Biotech, Beijing, China). Each sample included three technical replications.

## 5. Conclusions

We identified 41 candidate *AktDofs*, and many (39) of them were unevenly distributed on 15 high-quality assembled chromosomes of the *A. trifoliata* genome. All 41 *AktDofs* were classified into 7 groups, and in terms of evolution, they were mainly produced by WGD and had experienced a strong purifying selection. Of these *AktDofs*, 27 could be eudicot specific, and even *AktDof14* could be basal eudicot specific. Many *AktDofs* exhibited tissue- and developmental stage-specific expression patterns, although they were constitutively expressed at low levels. The evidence from the characteristics, expression profiles, and evolutionary experience suggested that *AktDofs* could have largely functional differences. We further identified four genes, namely, *AktDof21*, *AktDof20*, *AktDof36*, and *AktDof17*, that could be involved in the response to LD conditions, while the other three genes, namely, *AktDof26*, *AktDof16*, and *AktDof12*, could be involved in the response to darkness, which would overlap with the response to phytohormones. These results are very helpful for further research on the adaptation of *A. trifoliata* to environmental changes.

## Figures and Tables

**Table 1:** Characteristics of the identified *Dof* gene family members from the *A. trifoliata* genome.

Dof Genes	Gene Length (bp)	Number of Exons	Chromosome Location			Duplication Type	Putative Protein		
							Length (AA)	PI	MW (kDa)
*AktDof1*	607	2	chr1	9908538	9909145	Dispersed	192	8.97	21.70
*AktDof2*	488	2	chr1	24576861	24577349	WGD or Segmental	150	9.48	16.95
*AktDof3*	1355	1	chr1	26704731	26706086	WGD or Segmental	273	8.59	31.04
*AktDof4*	966	1	chr2	5412299	5413265	WGD or Segmental	194	8.94	21.30
*AktDof5*	1657	1	chr2	5509001	5510658	WGD or Segmental	308	8.71	33.27
*AktDof6*	833	1	chr2	5980914	5981747	WGD or Segmental	277	8.96	30.69
*AktDof7*	1438	1	chr3	43888045	43889483	WGD or Segmental	317	9.03	34.91
*AktDof8*	3242	2	chr3	44585557	44588799	WGD or Segmental	330	8.85	35.76
*AktDof9*	797	1	chr3	52617421	52618218	WGD or Segmental	265	9.33	29.27
*AktDof10*	942	1	chr4	1921930	1922872	Dispersed	273	4.73	30.57
*AktDof11*	782	1	chr4	10404006	10404788	WGD or Segmental	260	6.81	28.96
*AktDof12*	1388	1	chr4	10759535	10760923	Dispersed	308	9.11	32.57
*AktDof13*	1581	2	chr4	10814000	10815581	WGD or Segmental	332	9.23	36.12
*AktDof14*	1913	2	chr4	41906084	41907997	WGD or Segmental	313	9.14	34.43
*AktDof15*	2446	2	chr5	3830380	3832826	WGD or Segmental	301	6.04	33.46
*AktDof16*	3272	2	chr5	6188849	6192121	WGD or Segmental	469	6.65	51.93
*AktDof17*	1406	1	chr5	8819151	8820557	WGD or Segmental	297	6.75	33.39
*AktDof18*	2109	2	chr6	5604374	5606483	WGD or Segmental	287	7.11	32.07
*AktDof19*	2072	2	chr6	6865819	6867891	WGD or Segmental	343	9.05	36.62
*AktDof20*	3579	2	chr6	28715562	28719141	WGD or Segmental	489	5.98	53.72
*AktDof21*	2446	2	chr7	28997114	28999560	WGD or Segmental	306	6.26	34.39
*AktDof22*	3358	3	chr7	31830746	31834104	WGD or Segmental	522	8.56	57.10
*AktDof23*	1312	1	chr7	33314300	33315612	WGD or Segmental	245	6.43	27.86
*AktDof24*	2102	3	chr8	698486	700588	WGD or Segmental	318	8.92	34.53
*AktDof25*	1987	2	chr8	2192672	2194659	WGD or Segmental	284	7.59	31.81
*AktDof26*	3551	2	chr9	22881329	22884880	WGD or Segmental	489	6.19	54.40
*AktDof27*	2558	2	chr10	31290225	31292783	WGD or Segmental	285	8.49	31.33
*AktDof28*	2380	2	chr11	10569833	10572213	WGD or Segmental	286	8.12	31.21
*AktDof29*	2587	3	chr11	47368316	47370903	WGD or Segmental	287	8.76	31.85
*AktDof30*	878	1	chr11	47801725	47802603	WGD or Segmental	292	9.21	33.24
*AktDof31*	1676	1	chr12	3108299	3109975	Dispersed	213	8.46	23.45
*AktDof32*	1976	2	chr12	36036738	36038714	WGD or Segmental	261	9	28.81
*AktDof33*	1834	1	chr13	6715548	6717382	Tandem	285	9.07	31.45
*AktDof34*	2870	2	chr13	6735523	6738393	WGD or Segmental	308	8.65	34.15
*AktDof35*	639	1	chr13	9523741	9524380	WGD or Segmental	151	9.4	16.85
*AktDof36*	926	1	chr14	2280296	2281222	Dispersed	308	5.37	34.71
*AktDof37*	1590	2	chr14	3970765	3972355	WGD or Segmental	323	9.11	34.40
*AktDof38*	12,257	3	chr14	35699953	35712210	WGD or Segmental	480	5.32	52.39
*AktDof39*	1982	2	chr15	24196450	24198432	WGD or Segmental	307	8.03	34.09
*AktDof40*	1598	1	Contig00776	28846	30444	Dispersed	202	9.36	22.67
*AktDof41*	608	1	Contig01043	19276	19884	Dispersed	202	9.36	22.67

AA, amino acids; PI, isoelectric point; MW, molecular weight.

## Data Availability

All experiments and data are available in this article and the Supplementary Materials.

## Supplementary Materials

The following supporting information can be downloaded at: https://www.mdpi.com/article/10.3390/ijms24054973/s1.
