# Supplementary material for: Identification of Photoperiod- and Phytohormone-Responsive DNA-Binding One Zinc Finger (Dof) Transcription Factors in Akebia trifoliata via Genome-Wide Expression Analysis
Source: Int J Mol Sci. 2023 Mar 4;24(5):4973. doi: 10.3390/ijms24054973 (PMC10002981; doi:10.3390/ijms24054973)
Supplement: Supplementary file 1 [file ijms-24-04973-s001.zip › Supplementary Figure S1-S4.pdf]

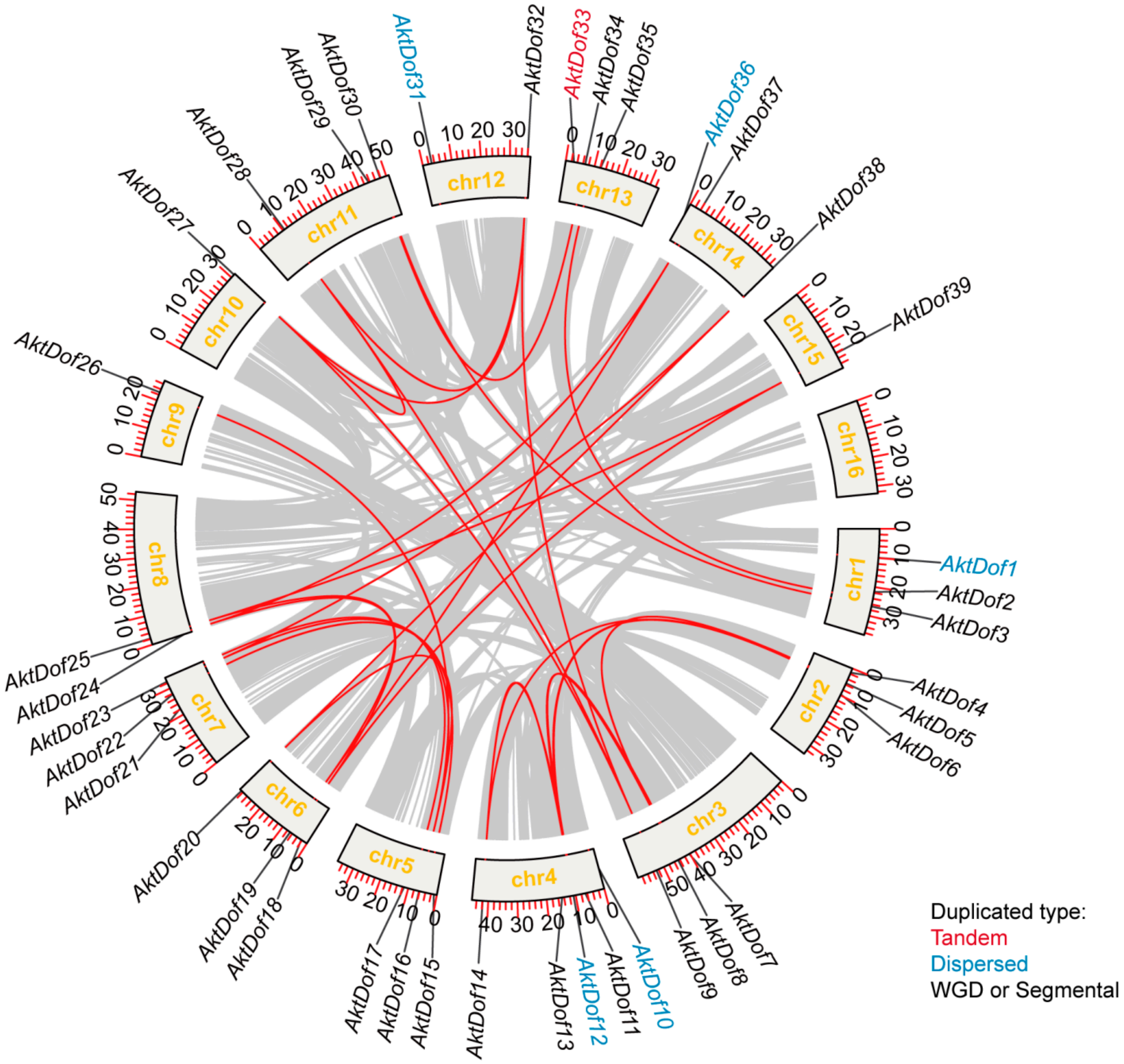

Figure S1. Intraspecies collinearity, replication events, gene clusters of *AktDofs*. WGD or segmental repeat genes are marked in black font, tandem repeat genes are marked in blue font, and proximal repeat genes are marked in red font. Gene clusters calculated by a 250kb sliding window are shown with the same symbols.

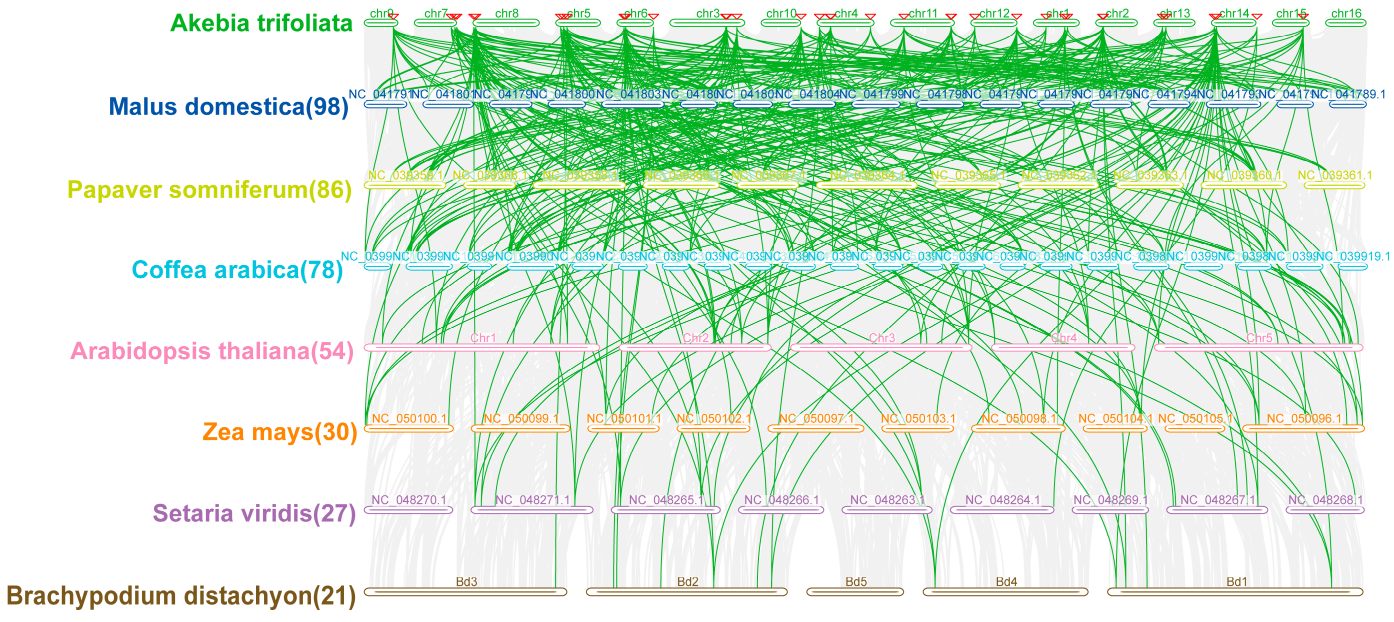

Figure S2. Collinearity analysis of *AktDofs* among two basal eudicots (*A. trifoliata* and *P. somniferum*), three core dicots (*A. thaliana*, *C. arabica*, *M. domestica*), and three monocots (*B. distachyon*, *S. viridis*, *Z. mays*). The green lines represent the Dof homologous gene pairs between the species and the *AktDofs*, the gray lines represent the genome-wide homologous gene pairs, and the collinear gene pairs are shown in parentheses. The number in parentheses after the species name represents the logarithm of collinear genes with *AktDofs*.

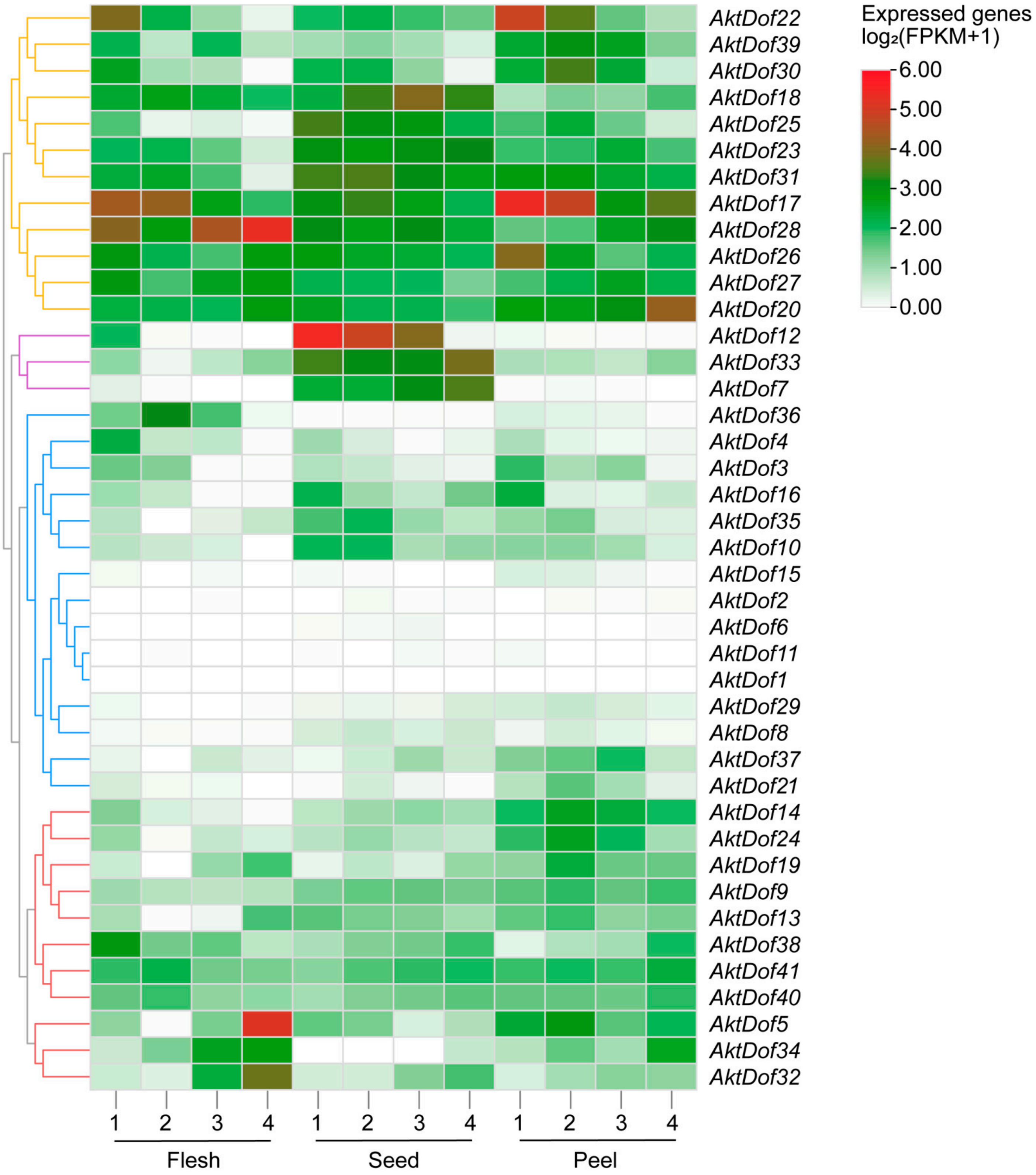

Figure S3. Expression profiles of 41 *AktDofs* in viarious tissues and organs. Cluster results of gene expressions, Fragments per kilobase of transcript per million fragmaents mapped (FPKM) values of *AktDofs* were transformed by log_2. White-green-red represent low to high relative enrichment of transcripts.

	AktDof22	*AktDof27*	*AktDof21*	*AktDof13*	*AktDof3*	*AktDof20*	*AktDof36*	*AktDof26*	*AktDof17*	*AktDof16*	*AktDof39*	*AktDof12*
0h	1.00											
LD 3h	1.03	36.90**	5.06 **	36.77	0.69	36.98**	55.07**	0.25	38.94**	1.03	0.33	3.58
LD 6h	0.51	4.53	0.57	4.47	0.42	18.22	12.73	0.15	6.84	0.24	0.16	1.86
LD 9h	0.71	6.36	0.80	13.46	0.72	15.75	11.00	0.05	6.32	0.40	0.44	3.25
LD 12h	0.40	7.77	0.24	10.12	0.21	8.76	2.86	0.13	0.98	0.95	0.24	3.04
LD 15h	0.61	20.75	1.37	55.67**	0.53	16.37	6.50	0.23	6.35	8.60	0.73	9.00
LD 18h	0.53	0.41	0.48	0.13	0.69	0.10	1.44	1.07	0.26	6.14	0.19	10.64**
LD 21h	0.38	0.41	0.26	0.32	1.13	0.50	3.21	2.58 **	0.24	15.21**	0.58	7.39
LD 24h	1.00	2.48	1.29	0.86	0.69	0.94	1.00	0.96	1.10	1.26	1.42	1.08
SD 3h	0.46	17.34**	0.55	9.46 **	0.30	0.49	1.43	0.21	8.79	0.22	1.72	0.12
SD 6h	0.09	13.46	0.15	5.27	0.33	0.37	1.39	0.05	4.20	0.05	0.26	0.07
SD 9h	0.42	13.81	0.13	7.79	0.85	0.37	2.69 **	0.18	12.56**	0.13	0.94	0.10
SD 12h	0.12	0.48	0.09	8.25	0.47	1.69	0.10	0.75	0.47	1.79	0.28	0.42
SD 15h	1.14	3.05	0.15	3.16	2.12	10.01**	0.12	4.18 **	0.73	2.44 **	1.73	2.67 **
SD 18h	0.20	0.50	0.20	0.95	0.63	1.76	0.15	0.57	0.21	0.34	0.97	0.60
SD 21h	0.41	1.00	0.11	0.47	0.34	2.71	0.07	0.64	0.29	0.57	0.55	0.32
SD 24h	0.61	1.05	0.56	2.09	0.68	4.84	0.68	3.36	1.03	1.14	1.37	1.80

ZT(h)

Figure S4. Expression pattern of 12 *AktDofs* in different photoperiod processing. Colored undertones represent peaks with significant differences. Data are the average of three replicates, statistical significance was determined by a two-sided t-test. * represent the difference between peak expression and 0h (**, p < 0.01). Time (h) is expressed as hours from dawn (ZT, zeitgeber), and black represents night. The top is LD processing, the bottom is SD processing.
